# Trauma After Cochlear Implantation: The Accuracy of Micro–Computed Tomography and Cone-Beam Fusion Computed Tomography Compared With Histology in Human Temporal Bones

**DOI:** 10.1097/MAO.0000000000003835

**Published:** 2023-02-22

**Authors:** Matti Iso-Mustajärvi, Tuomo Silvast, Tuomas Heikka, Jyrki Tervaniemi, Roger Calixto, Pia H. Linder, Aarno Dietz

**Affiliations:** ∗ Department of Otorhinolaryngology, Kuopio University Hospital; † SIB Labs Infrastructure Unit, Faculty of Science and Forestry, University of Eastern Finland; ‡ Department of Radiology, Kuopio University Hospital, Kuopio, Finland; § Research and Technology, Oticon Medical, France

**Keywords:** 3D image fusion, Array, Cochlear implants, Cone-beam tomography, Electrode, Histology, Intracochlear trauma, Micro-CT, Temporal bone

## Abstract

Micro-computed tomography (micro-CT) and cone-beam computed tomography (CBCT), in conjunction with the image fusion technique, may provide similar results for trauma assessment after cochlear implantation, with respect to the trauma evaluation in preclinical cochlear implant (CI) studies, as the histology.

**Background:**

Before clinical use, novel cochlear implant (CI) designs are tested in temporal bone (TB) studies for usability and risk evaluation. The criterion standard for evaluating intracochlear insertion trauma and electrode location has historically been with histological samples. Progress of modern imaging technology has created alternatives to classic histology. This study compares the micro-CT and CBCT fusion images between histological samples in a preclinical CI study.

**Methods:**

Fourteen freshly frozen TBs were inserted with a lateral wall research CI electrode. All TBs were scanned with CBCT preoperatively and postoperatively. After insertion, the TBs were prepared for micro-CT and histology. Twelve TBs underwent first a micro-CT and then the histological process. The CBCTs were used for image fusion, and all three different methods were used for intracochlear trauma evaluation. The results were compared between methods.

**Results:**

There were 4 of 14 translocations detected with the fusion image method and 3 of 12 with the micro-CT and histology. When compared, the trauma grades converged and were not statistically significant.

**Conclusion:**

The trauma grading based on micro-CT is comparable to the histology. The image fusion technique based on CBCT is less accurate because it relies on an empirical assumption of the basal membrane localization, but it is clinically applicable.

## INTRODUCTION

The preservation of the delicate intracochlear structures during cochlear implant (CI) surgery provides the best possible prerequisite for successful hearing rehabilitation with CIs (1,2). In addition to the surgical technique, the electrode design has a major effect on the likelihood of intracochlear trauma (1,3). For the preclinical evaluation of novel electrode arrays, studies on human temporal bones (TBs) are usually carried out for the preclinical evaluation of the new electrode arrays, as these are mandatory for approval by the authorities. In addition, TB studies provide valuable data on the insertion characteristics of novel arrays in a near real-life setting, however, keeping in mind the differences compared with in vivo implantation.

A review conducted by Dhanasingh and Jolly (3) reported that modern lateral wall (LW) or straight electrodes cause less trauma than precurved or modiolar electrodes. The softer and thinner the electrode, the less is the likelihood for causing inner trauma, such as scalar translocation. However, if the electrode is too flexible, it may be difficult to insert, ending up with partial inserted arrays that do not adequately cover the spiral ganglions along the cochlear duct, thus compromising the hearing results (4,5). Therefore, stiffer arrays may be favored by CI surgeons because they are easier to insert but have the disadvantage of causing trauma more easily (6,7).

Currently, histology is considered the “criterion standard” for trauma evaluation in TB studies. Histology enables for a reliable trauma evaluation because it provides a detailed visualization of cochlear structures such as the basilar membrane (BM). However, it is very time-consuming and technically challenging to perform accurately. Furthermore, the interpretation of the histological results may be impaired because of several factors: electrode movements can occur during the drilling out of the cochlea and during the manipulation of the specimen; the fixation of the specimen can be troublesome because uneven distribution of the fixation solution and epoxy causes air inclusions, which hamper the quality of image and preclude a reliable trauma assessment.

Cone-beam computed tomography (CBCT) has established its position as a clinical modality for postoperative CI imaging. It has also been applied for CI studies in TB (8–11). Several studies have shown that the accuracy of trauma assessment can be significantly improved with the image fusion technique of preimplant and postimplant CBCT images (12–14).

Micro–computed tomography (micro-CT) is another feasible imaging modality used for the investigation of cochlear trauma in TB studies (15–17). It can provide highly detailed images of the cochlea, it is significantly less time-consuming, and it requires no manipulation when compared with histology. Micro-CT also provides visualization of the electrode location inside the cochlea via three-dimensional (3D) reconstructions of the electrode and inner ear structures. An important advantage of micro-CT over histology is that the objects can be viewed in multiple different planes and at different angles.

The aim of this study was to compare the accuracy of preimplant and postimplant fusion imaging with micro-CT and histology. The hypothesis was that micro-CT and CBCT in conjunction with the image fusion technique would provide similar results for trauma assessment after cochlear implantation, regarding the trauma evaluation in preclinical CI studies, as the histology.

A secondary aim was to evaluate surgical handling and insertion results of the research electrode with a modified stiffness profile with special attention to whether the increase in basal stiffness would prevent proximal trauma at the expense of an increased risk for apical trauma.

## MATERIALS AND METHODS

We collected 14 freshly frozen TBs for this study. The study had institutional approval and fulfilled the Helsinki Declaration for the ethical use of human material. There were seven right- and seven left-sided bones with normal anatomy (Table 1). A CI electrode was inserted into the cochlea of each TB, through the round window using soft surgery techniques.

**TABLE 1:** Descriptive data and overall trauma grading between different methods

TB	Side	A-Measure (mm)	B-Measure (mm)	Basal Length (mm)	IDA	Trauma Grading		
						CBCT Fusion	Micro-CT	Histology
TB01	Left	9.5	6.8	8.6	397	0	1A	1A
TB02	Right	9.2	6.7	8.5	446	3	3	2
TB03	Left	9.3	7.0	8.5	407	1	1A	1A
TB04	Right	10.1	7.4	8.8	397	1	1A	1A
TB05	Left	9.5	7	8.4	407	3	3	3
TB06	Left	9.6	6.8	8.9	401	1	1B	1B
TB07	Right	9.5	7.2	8.5	396	3	N.A.	N.A.
TB08	Right	9.6	7.2	8.6	413	1	1A	1A
TB09	Left	9.4	6.4	8.5	442	1	1B	1B
TB10	Right	9.1	6.5	8.6	499	1	1A	1A
TB11	Left	9.0	6.1	7.5	493	3	3	3
TB12	Right	8.9	6.7	7.9	381	1	1B	1B
TB13	Right	9.5	7.3	8.2	451	1	N.A.	N.A.
TB14	Left	9.7	7.5	8.6	395	1	1B	1A
Mean		9.4	6.9	8.4	423			

CBCT indicates cone-beam computed tomography; IDA, insertion depth angle; micro-CT, micro-computed tomography; TB, temporal bone.

### Electrode

For this study, we approached Oticon Medical (Oticon Medical, Copenhagen, Denmark) to create a research electrode array (REA) that increased the chances of traumaticity in the first turn. They provided a research array based on the EVO platform (LW array with 20 full-band contacts, 25 mm long) with a modified stiffness profile by using a thicker contact wire that spans the six most basal contacts, making it less flexible for the basal 8 mm of the array. As with the EVO, the REA has two conical push rings at the base for sealing the cochleostomy opening and for the handling of the electrode.

### Surgery

All insertions were performed according to the institutions best practice for hearing preservation surgery. A transmastoid posterior tympanotomy approach was used in all TBs. The round-window niche was exposed with a 1.5-mm diamond burr by removing the bony overhang. The round-window membrane was carefully opened before the insertion with a hypodermic needle. All insertions were performed by the author (A.D.). Each insertion was recorded via an operating microscope, and the surgeons' feedback was documented after each insertion. The electrode was fixed to the facial recess with cyanoacrylate glue after the insertion.

### CBCT and Micro-CT

All 14 TBs were scanned with the CBCT (ProMax 3D Max; Planmeca Oy, Helsinki, Finland) preoperatively and immediately after insertion. The preinsertion scans were performed using the following parameters: tube voltage, 80 kV; tube current, 16 mA; imaging time, 15 seconds; and field of view, 50 × 55 mm. The respective postinsertion scan parameters were 96 kV, 7 mA, 15 seconds, and 50 × 55-mm field of view. Axial, sagittal, and coronal slices with 150-mm isometric voxel size were reconstructed using Planmeca Romexis software. For the postinsertion scan, a metal artifact removal algorithm (ARA by Romexis) was applied.

A- and B-measurements, as well as the length of the basal turn from RWM to the LW, were measured from the preoperative images according to Escudé et al. (18). The insertion depth angle (IDA) was measured from the postoperative CBCT images. All of the measurements were done independently by three authors (M.I.-M., A.D., and P.H.L.), and the average of these measurements was used for the analysis. The descriptive data are summarized in Table 1.

After postoperative CBCT scans, the TB was further processed; the TB was reduced to only the otic capsule for micro-CT and histological processing. The sample size was approximately 15 × 15 × 15 mm after trimming. Before the micro-CT scan, the stapes footplate was opened, and the perilymph was removed. Care was taken not to interfere with electrode position during the perilymph removal.

The micro-CT scans were performed with a Skyscan 1172 (Bruker microCT, Kontich, Belgium). Scan parameters were the following: 100 kV, 100 μA, Al + Cu filter equivalent to 2 mm Al, and 180-degree rotation with 0.15-degree step. A pixel size of 4.5 μm was used. For the evaluation of the micro-CT images, we used the Dragonfly software (ORS, Quebec, Canada). Three-dimensional reconstructions and supplementary videos were processed using NRecon, CT Analyzer, and CT Volume programs (Bruker micro-CT). The trauma assessment was done independently by the two authors (A.D. and M.I.-.M.).

### Image Fusion

Preimplant and postimplant CBCT images were fused with a commercially available image fusion software iPlan (iPlan Net 3.6.0 Build 77; BrainLab AG, Munich, Germany). The image fusion method for CI imaging has been previously described in detail (12,13). The electrode reconstruction was made by thresholding based on the Hounsfield unit values. Obvious artifacts were removed manually. The reconstructed electrode was then projected onto the preoperative images to provide artifact-free images with the electrode in place.

### Histology

After micro-CT, the cochleae were briefly immersed in 70% ethanol solution. Dehydration of samples was carried out with ascending concentrations of ethanol. Polymethylmethacrylate (PMMA) was used for the final embedding of the samples. For both the dehydrating process and embedding with PMMA, a mild vacuum was used to ensure the infiltration of solutions. For the analysis, the PMMA blocks were ground and polished for imaging through a stereo microscope with a digital camera. The whole cochlea was ground with images taken every 200 to 500 μm for the analysis.

### The Electrode Placement and Classification of Trauma

Electrode location was determined for IDA 90, 180, 270, and 360 degrees and for the tip of the electrode. This was done for each method. With regard to the trauma grading, the Eshragi (19) scale was used for micro-CT and histology. For the fusion imaging, we used trauma grading described previously by Sipari et al. (13).

### Statistical Analysis

The comparison was made with Wilcoxon signed rank test and *χ*^2^ test. Correlations were analyzed with the Pearson test. Statistical test was performed with the Statistical Packages for the Social Sciences (SPSS) for Windows version 25 (SPSS Inc., Chicago, IL). For the comparison between trauma grades, the Eshragi grades 1A and 1B were interpreted as grade 1 in micro-CT and histological samples to have equivalent data with fusion images.

## RESULTS

In all TBs, the insertion could be easily performed through the round window without any noticeable resistance during the insertion process. All insertions could be carried out easily, and there was no need to pull back and reinsert in any TB. Full insertion with all contacts inside the cochlea was achieved in 12 of the 14 TBs. Two electrode arrays exhibited partial insertions: in TB07, there was one extracochlear contact, and in TB12, there were two extracochlear contacts. We did not notice any significant electrode bulging outside the cochlea in any of the insertions. The mean IDA measured from the CBCT images was 423 degrees (range, 381–499 degrees) in all of the 14 TBs.

Trauma assessment via the CBCT fusion image technique showed scala dislocation in four TBs (TB02, TB05, TB07, and TB11; 29%). In TB02, the electrode translocated occurred at IDA 360 degrees and the total insertion depth was 446 degrees. In TB05, TB07, and TB11, translocation occurred at IDA 180 degrees. The trauma grading is summarized in Table 1. Table 2 shows the comparison between trauma grading with the fusion imaging, micro-CT and histology.

**TABLE 2:** Distribution of insertion trauma with respect to different examination points regarding the insertion depth angle

TB	90°			180°			270°			360°			Tip Region		
	FI	μCT	Histology	FI	μCT	Histology	FI	μCT	Histology	FI	μCT	Histology	FI	μCT	Histology
TB01	0	0	0	0	0	0	0	0	0	0	0	0	0	1	1
TB02	0	0	0	0	1	1	1	0	0	1	3	3	3	3	2
TB03	0	0	0	0	1	1	0	0	0	1	0	0	1	1	1
TB04	0	0	0	1	0	1	0	1	1	0	1	1	0	1	1
TB05	0	0	0	3	3	3	3	2	2	3	2	2	3	2	2
TB06	0	0	0	0	1	1	1	1	1	1	0	0	0	0	0
TB08	0	0	0	0	0	1	1	0	0	0	1	0	0	1	1
TB09	0	0	0	1	1	1	0	1	1	0	1	1	1	1	1
TB10	0	0	0	0	1	1	1	1	1	1	1	1	1	1	1
TB 11	1	0	0	3	3	3	2	2	2	0	1	1	1	1	1
TB 12	1	0	0	1	1	1	0	1	1	0	1	1	0	1	1
TB 14	0	0	0	0	1	1	0	1	1	1	1	1	1	1	1

TB02 and TB13 are excluded because of missing μCT and histological results.

FI, fusion image technique; TB, temporal bone; μCT = micro-CT.

TB07 and TB13 were excluded from the micro-CT and histological analysis because of displacement of the electrode arrays during the preparation of the specimen. For the remaining 12 TBs, both micro-CT and histology were of adequate quality, and the Eshragi trauma scaling could be applied (image 1). In the micro-CT evaluation, trauma scaling for TB02 was class 3 (“translocation”), whereas in the histological analysis, trauma grading was class 2 (“rupture of basal membrane”). In the remaining TBs, trauma grading was identical for micro-CT and histology (Table 1).

There was no correlation between the IDA and trauma (*r* = 0.216, *p* = 0.458), A-measure and trauma (*r* = −0.462, *p* = 0.093), or the basal length and trauma grading (*p* = 0.220, *r* = 0.450).

All of the methods provided similar results for trauma evaluation. There was a good agreement of trauma grading with all methods. The interpretation between micro-CT and histology was almost identical; a statistically significant difference could not be found (*p* = 1.000). We found no statistical significance in trauma grading between either of the methods: fusion imaging and micro-TT (*p* = 0.133) or the fusion imaging and histology (*p* = 0.148).

## DISCUSSION

**FIG. 1:**
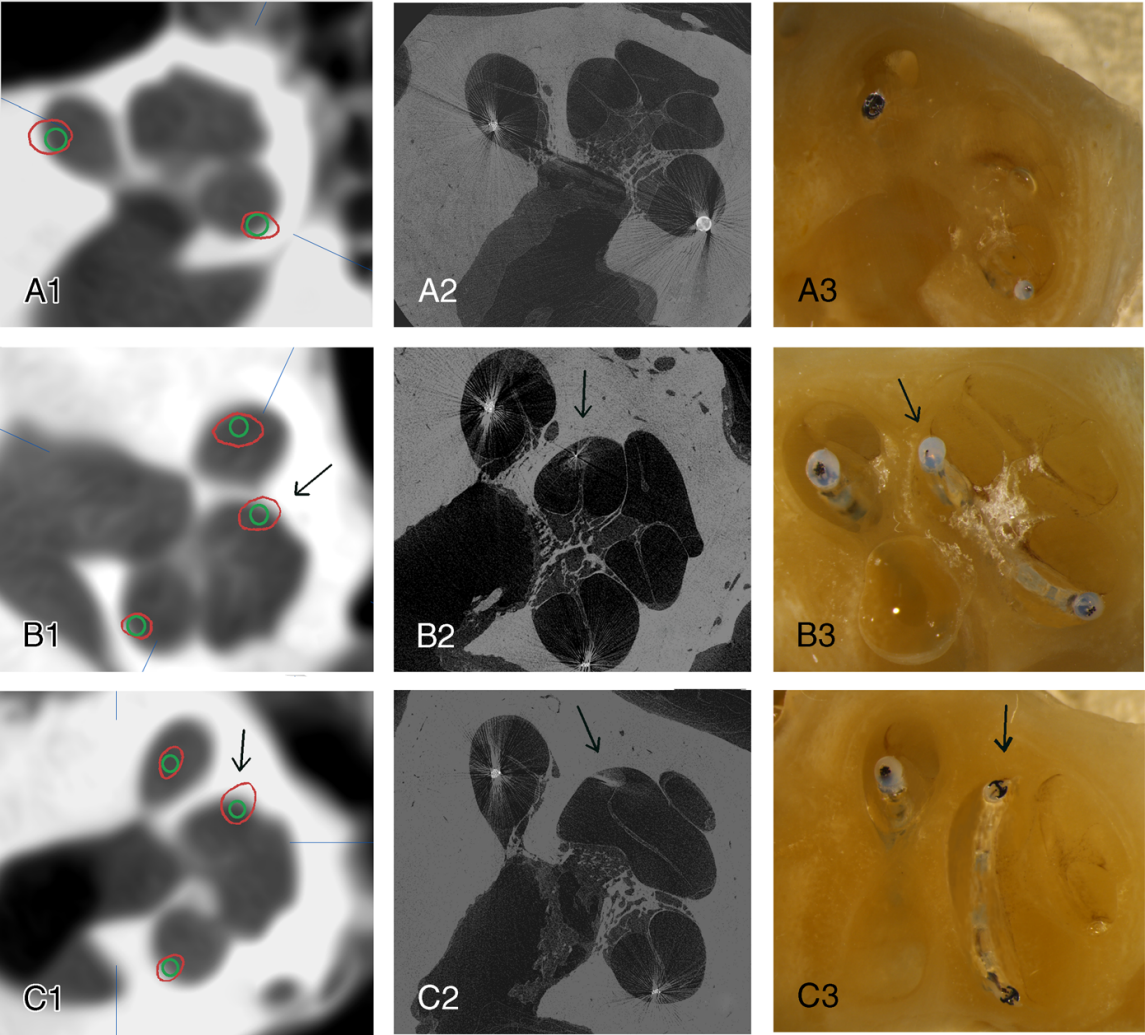
CBCT 3D fusion, micro-CT, and histological images (1, 2, and 3, respectively) of TB14 (*A*), TB9 (*B*), and TB2 (*C*). TB14 shows no trauma, and TB9 shows lifting at the tip region (black arrow). TB2 has dislocation in C1 and C2 images pointed by black arrow. In C3, the trauma was interpreted as rupture (pointed by black arrow). 3D indicates three-dimensional; CBCT, cone-beam computed tomography; micro-CT, micro-computed tomography; TB, temporal bone.

The electrode placement plays a crucial part in the success of CI surgery. During the development of new electrodes, histology has been the method for electrode placement evaluation in TB studies. Constant progress in imaging technology, accuracy, and use of modern 3D reconstruction techniques are able to provide fast and reliable information regarding the intracochlear placement of electrodes. However, applying the Eshragi trauma scaling in radiological data is often challenging, and thus, histology is still considered as the criterion standard for trauma evaluation. In this study, we aimed to develop more accurate imaging methods for evaluation of the insertion results in TB studies and exploit the possibilities of the 3D reconstructions (Fig. 1).

**FIG. 2:**
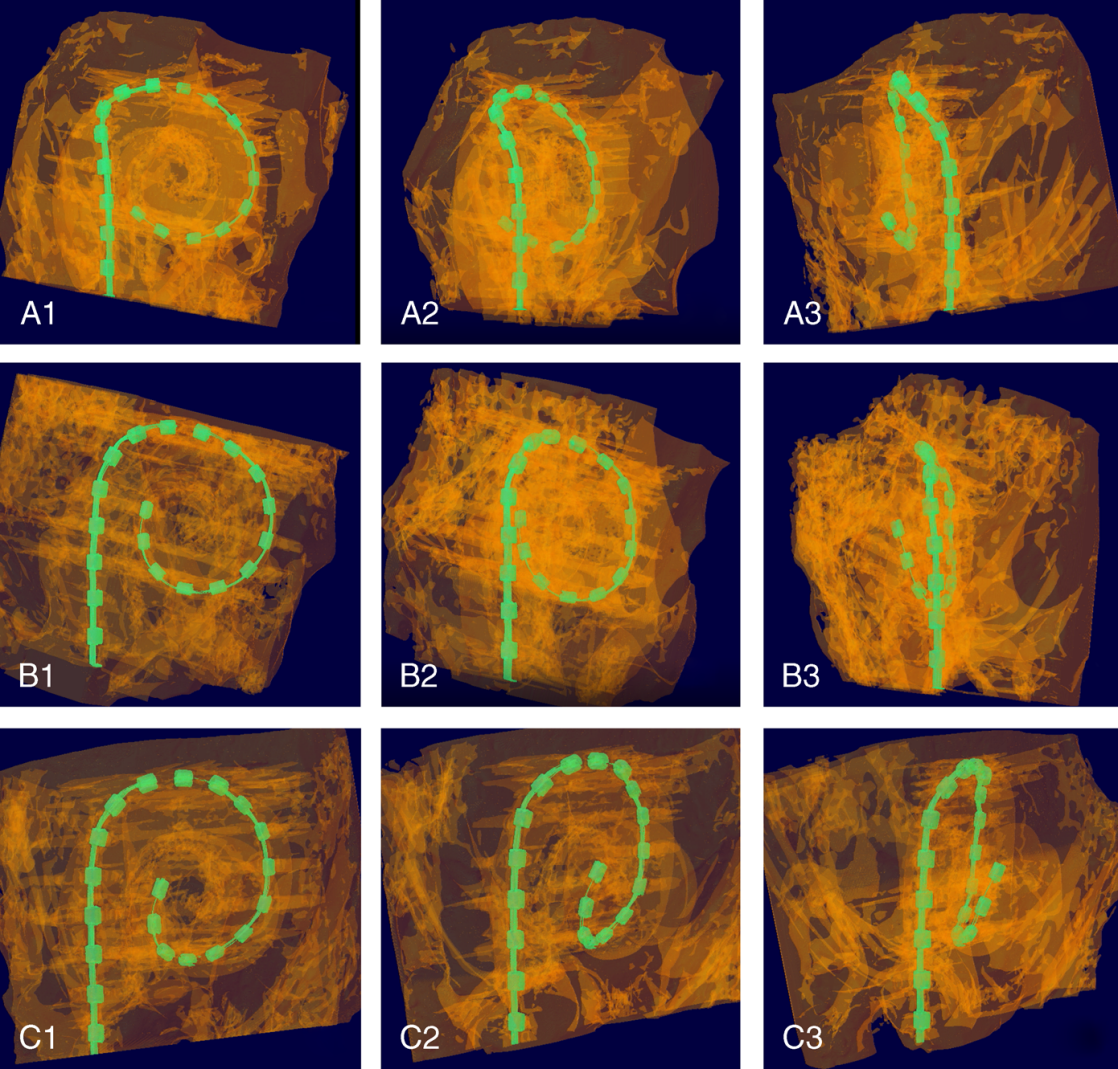
Images from 3D reconstructions videos of TB14 (A1, A2, and A3), TB9 (B1, B2, and B3), and TB2 (C12, C2, and C3). Videos can be found from supplementary e-material, http://links.lww.com/MAO/B576, http://links.lww.com/MAO/B577, http://links.lww.com/MAO/B578. 3D indicates three-dimensional; TB, temporal bone

Micro-CT offers significant advantages over histology in TB studies: micro-CT is a nondestructive imaging method and considerably faster to perform (micro-CT 4–9 h and histology approximately 90 d). In modern micro-CT devices, detectors are even faster and more sensitive, and hence, imaging times can be even shorter. The development of more elaborated reconstruction algorithms also shortens the processing time of obtained images. Thus, accurate micro-CT images can be available in a few hours. Another advantage of the micro-CT is that the evaluation can be performed in a step-by-step “film strip,” following the whole length of electrode inside the cochlea (17). In addition, the micro-CT provides the possibility for 3D reconstructions, from which the exact placement of electrodes inside the scala tympani can be seen more easily (Fig. 2). Subtle anatomical variations in the cochlear duct, such as the so-called rollercoaster duct form, are only visible and recognizable from the 3D reconstructions. Two-dimensional images, histological or radiological, cannot convey that information.

However, we noted discrepancy between micro-CT and histology grading in TB02, in which micro-CT classified trauma as grade 3 (translocation) and histology as grade 2 (rupture of the BM). However, clinically both conditions could be regarded as inner ear trauma with probably similar clinical outcome, as it is likely that, in both situations, any residual hearing would have been lost. The separation between the elevation of BM and rupture of BM with current imaging can be most challenging and even impossible, despite advanced reconstruction models (16) and image fusion.

Metal artifacts may reduce the micro-CT image quality, and the artifact pattern is difficult to predict. Removing the perilymph before imaging improves the contrast between BM, scala vestibuli, and tympani, and aids the assessment of trauma with the CBCT image fusion technique and with micro-CT (20,21). The micro-CT provides better resolution compared with CBCT, but imaging time is longer than with CBCT. The sample size with micro-CT is limited to smaller objects than with CBCT, as well.

Earlier studies have shown that micro-CT is feasible for in vitro electrode studies (15–17). Whereas Le Breton et al. (15) concluded that micro-CT is not sufficiently accurate for the reliable assessment of insertion trauma, Postnov et al. (17) found nearly equal visualization of the cochlear structures and the electrode array as in histological samples. Similar to our study, Postnov et al. removed the perilymph to increase the contrast of the BM and better visualization. Teymouri et al. (16) found no difference in the electrode localization in 17 TBs assessed with micro-CT and histology. Teymouri et al. (16) classified electrode location as scala tympani if the array was fully inside the scala tympani, and as scala vestibuli if the electrode was above Reissner's membrane and intermediate for electrodes at the scala media region. They also chose to use a grading differing from the Eshragi scale, as in their 3D reconstruction model, it was difficult to separate the elevation and rupture (16).

The currently used trauma grading is based on histology and thus not directly applicable to radiological evaluation. Therefore, a new trauma grading applicable for imaging should be developed. Mosnier et al. (9) and De Seta et al. (8) used a simplified trauma staging (no trauma; dislocation) in their image-based studies. Accordingly, our study supports the feasibility of micro-CT for trauma assessment, with nearly the same accuracy as histology. However, there is a need for more validation studies to establish the value of micro-CT for electrode studies, as it has been rarely exploited in TB studies. To further evaluate the feasibility of micro-CT for CI TB studies, more research is needed including the development of a reliable trauma grading scale.

For the CBCT fusion image technique, differences in trauma grading are explained by the variety of the localization of the BM, which cannot be visualized with CBCT. Most of the differences were between grades 0 and 1. The clinical relevance of “no trauma” (grade 0) compared with the “touching of the BM” (grade 1) is not well distinct. There was only one single location in one TB when the CBCT image fusion technique graded the trauma as grade 1 compared with major trauma (grade 2 or 3) graded in the micro-CT and histology (TB02, 360-degree point). Nevertheless, the overall trauma grade in TB02 was 3 for the image fusion technique, and the grades for micro-CT and histology were 3 and 2, respectively. The accuracy of the image fusion technique has been documented in our previous studies (12,13). This present study again verifies the overall good accuracy of the fusion image technique for TB studies, although the main advantage is its clinical feasibility.

With respect to the characteristics of the REA, the trauma rate (REA; 29% in fusion image and 25% for micro-CT and histology, including the ruptured BM) is comparable to results obtained with the EVO electrode (13). The IDA for the REA in this study was almost identical with the previous study, 423 degrees (range, 381–499 degrees) versus 416 degrees (range, 368–501 degrees), respectively. Therefore, it seems that the increased basal stiffness did not facilitate deeper insertions. Even small alterations to the electrode array design, such as a slightly stiffer basal section, may influence in insertion properties of the electrode array. Thus, the suitability of new designs should be evaluated in TB studies in advance of clinical use.

Main limitations of this study are the small sample sizes. The use of single electrode is also a limitation; thus, the different electrodes may cause different size of artifacts, so our findings have to be confirmed for other types of electrodes and manufacturers.

The strengths of this study are the availability of all three different methods for electrode location evaluation. Our institution has standardized methods for electrode studies and experience with different electrode characteristics. Thus, the presented results are comparable to previous studies.

## CONCLUSION

This study supports the feasibility of micro-CT for trauma assessment, with nearly identical accuracy as histology. Although micro-CT provides very accurate information on the scalar location of the electrode, it is not sufficiently sensitive for the differentiation between BM elevation and rupture and therefore cannot completely substitute histology for the assessment of insertion trauma. The image fusion technique based on CBCT is less accurate because it relies on an empirical assumption of the basal membrane localization, but it is clinically applicable.

## Supplementary Material
