# Supplementary material for: Trauma After Cochlear Implantation: The Accuracy of Micro–Computed Tomography and Cone-Beam Fusion Computed Tomography Compared With Histology in Human Temporal Bones
Source: Otol Neurotol. 2023 Feb 22;44(4):339–45. doi: 10.1097/MAO.0000000000003835 (PMC10022666; doi:10.1097/MAO.0000000000003835)
Supplement: Supplementary file 1 [file on-44-339-s001.docx]

**Trauma after cochlear implantation: The accuracy of micro-CT and cone-beam fusion CT compared to histology in human temporal bones.**

**Abstract**

**Hypothesis:** Micro-computed tomography (micro-CT) and cone beam computed tomography (CBCT), in conjunction with the image fusion technique may provide similar results for trauma assessment after cochlear implantation, with respect to the trauma evaluation in preclinical cochlear implant studies, as the histology.

**Background:** Before clinical use, novel cochlear implant (CI) designs are tested in temporal bone (TB) studies for usability and risk evaluation. The gold standard for evaluating intracochlear insertion trauma trauma and electrode location has historically been with histological samples. Progress of modern imaging technology has created alternatives to classic histology. This study compares the Micro-CT and CBCT fusion images between histological samples in a preclinical CI study.

**Methods:** 14 freshly frozen TBs were inserted with a lateral wall research CI electrode. All TBs were scanned with CBCT pre- and postoperatively. After insertion the TBs were prepared for micro-CT and histology. Twelve TBs underwent first a micro-CT and then the histologic process. The CBCTs were used for image fusion and all three different methods were used for intra cochlear trauma evaluation. The results were compared between methods.

**Results:** There were 4/14 translocations detected with the fusion image method and 3/12 with the micro-CT and histology. When compared, the trauma grades converged and were not statistically significant.

**Conclusion:** The trauma grading based on micro-CT is comparable to the histology. The image fusion technique based on CBCT is less accurate as it relies on an empirical assumption of the basal membrane localization, but it is clinically applicable.

**Introduction**

The preservation of the delicate intracochlear structures during cochlear implant surgery provides the best possible prerequisite for successful hearing rehabilitation with cochlear implants (CI) (1, 2). In addition to the surgical technique, the electrode design has a major effect on the likelihood of intracochlear trauma (1, 3). For the preclinical evaluation of novel electrode arrays, studies on human temporal bones (TB) are usually carried out for the preclinical evaluation of the new electrode arrays, as these are mandatory for approval by the authorities. In addition, TB studies provide valuable data on the insertion characteristics of novel arrays in a near real-life setting, however, keeping in mind the differences compared to in-vivo implantation.

A review conducted by Dhanasingh et al. (3) reported that modern lateral wall (LW) or straight electrodes cause less trauma than pre-curved or modiolar electrodes. The softer and thinner the electrode, the less is the likelihood for causing inner trauma trauma, such as scalar translocation. However, if the electrode is too flexible, it may be difficult to insert ending up with partial inserted arrays which do not adequately cover the spiral ganglions along the cochlear duct thus compromising the hearing results (4, 5). Therefore, stiffer arrays may be favoured by CI surgeons because they are easier to insert but have the disadvantage of causing trauma more easily (6, 7).

Currently, histology is considered the ‘gold standard’ for trauma evaluation in TB studies. Histology enables for a reliable trauma evaluation as it provides a detailed visualization of cochlear structures such as the basilar membrane. However, it is very time consuming and technically challenging to perform accurately. Furthermore, the interpretation of the histologic results may be impaired due to several factors: electrode movements can occur during the drilling out of the cochlea as well as during the manipulation of the specimen; the fixation of the specimen can be troublesome as uneven distribution of the fixation solution and epoxy cause air inclusions which hamper the quality of image and preclude a reliable trauma assessment.

Cone-beam computed tomography (CBCT) has established its position as a clinical modality for postoperative CI imaging. It has also been applied for CI studies in TB (8-11). Several studies have shown that the accuracy of trauma assessment can be significantly improved with the image fusion technique of pre-and post-implant CBCT images (12-14).

Micro-Computed Tomography (micro-CT) is another feasible imaging modality used for the investigation of cochlear trauma in TB studies (15-17). It can provide highly detailed images of the cochlea, it is significantly less time consuming and requires no manipulation when compared to histology. Micro-CT also provides visualization of the electrode location inside the cochlea via 3D reconstructions of the electrode and inner ear structures. An important advantage of micro-CT over histology is that the objects can be viewed in multiple different planes and at different angles.

The aim of this study was to compare the accuracy of pre-implant and post-implant fusion imaging with micro-CT and histology. The hypothesis was that micro-CT and CBCT in conjunction with the image fusion technique would provide similar results for trauma assessment after cochlear implantation, regarding the trauma evaluation in preclinical cochlear implant studies, as the histology.

A secondary aim was to evaluate surgical handling and insertion results of the research electrode with a modified stiffness profile with special attention to whether the increase in basal stiffness would prevent proximal trauma at the expense of an increased risk for apical trauma.

**Materials and Methods**

We collected 14 freshly frozen TBs for this study. The study had institutional approval and fulfilled the Helsinki Declaration for the ethical use of human material. There were seven right and seven left-sided bones with normal anatomy (Table 1). A CI electrode was inserted into the cochlea of each TB, through the round window using soft surgery techniques.

**Electrode**

For this study we approached Oticon Medical (Oticon Medical, Copenhagen, Denmark) to create a research electrode array (REA) that increased the chances of traumaticity in the first turn. They provided a research array based on the EVO platform (LW array with 20 full band contacts, 25mm long) with a modified stiffness profile by using a thicker contact wire that spans the 6 most basal contacts making it less flexible for the basal 8 mm of the array. As with the EVO, the REA has two conical push rings at the base for sealing the cochleostomy opening and for the handling of the electrode.

**Surgery**

All insertions were performed according to the institutions best practice for hearing preservation surgery. A transmastoid posterior tympanotomy approach was used in all TBs. The round window niche was exposed with a 1.5 mm diamond burr by removing the bony overhang. The round window membrane was carefully opened prior to the insertion with a hypodermic needle. All insertions were performed by the author (AD). Each insertion was recorded via an operating microscope and the surgeons feedback was documented after each insertion. The electrode was fixed to the facial recess with cyanoacrylate glue after the insertion.

**Cone-Beam Computed Tomography and Micro-Computed Tomography**

All 14 temporal bones were scanned with the CBCT (ProMax 3D Max, Planmeca Oy, Helsinki, Finland) preoperatively and immediately after insertion. The pre-insertion scans were performed using the following parameters: tube voltage 80 kV, tube current 16 mA, imaging time 15 seconds, and FOV 50 x 55 mm. The respective post insertion scan parameters were 96 kV, 7 mA, 15 seconds, and 50 x 55 mm FOV. Axial, sagittal, and coronal slices with 150-mm isometric voxel size were reconstructed using Planmeca Romexis software. For the post-insertion scan, a metal artefact removal algorithm (ARA by Romexis) was applied.

A- and B-measurements, as well as the length of the basal turn from RWM to the lateral wall were measured from the preoperative images according to Escudé (18). The insertion depth angle (IDA) was measured from the postoperative CBCT images. All of the measurements were done independently by three authors (MI-M, AD and PL), and the average of these measurements were used for the analysis. The descriptive data is summarized in Table 1.

After postoperative CBCT scans, the TB was further processed; the temporal bone was reduced to only the otic capsule for micro-CT and histological processing. The sample size was approximately 15 x 15 x 15 mm after trimming. Before the micro-CT scan, the stapes footplate was opened, and the perilymph was removed. Care was taken not to interfere with electrode position during the perilymph removal.

The micro-CT scans were performed with a Skyscan 1172 (Bruker microCT, Kontich, Belgium). Scan parameters were the following: 100 kV, 100 µA, Al+Cu filter equivalent to 2 mm Al, and 180° rotation with 0.15° step. A pixel size of 4.5 µm was used. For the evaluation of the micro-CT images, we used the Dragonfly software (ORS, Quebec, Canada). 3D reconstructions and supplementary videos were processed using NRecon, CT Analyzer, and CT Volume programs (Bruker micro-CT). The trauma assessment was done independently by the two authors (AD and MI-M).

**Image Fusion**

Pre- and post-implant CBCT images were fused with a commercially available image fusion software iPlan (iPlan Net 3.6.0 Build 77, BrainLab AG, Munich, Germany). The image fusion method for CI imaging has been previously described in detail (12, 13). The electrode reconstruction was made by thresholding based on the Hounsfield unit values (HU). Obvious artefacts were removed manually. The reconstructed electrode was then projected onto the preoperative images to provide artefact free images with the electrode in place.

**Histology**

After Micro-CT the cochleae were briefly immersed in 70 % ethanol solution. Dehydration of samples was carried out with ascending concentrations of ethanol. Polymethylmethacrylate (PMMA) was used for the final embedding of the samples. For both the dehydrating process and embedding with PMMA, a mild vacuum was used to ensure the infiltration of solutions. For the analysis, the PMMA blocks were ground and polished for imaging through a stereo microscope with a digital camera. The whole cochlea was ground with images taken every 200 to 500 µm for the analysis.

**The Electrode Placement and Classification of Trauma**

Electrode location was determined for IDA 90, 180, 270, 360 degrees and for the tip of the electrode. This was done for each method. With regard to the trauma grading, the Eshragi (19) scale was used for micro-CT and histology. For the fusion imaging, we used trauma grading described previously by Sipari et al. (13).

**Statistical Analysis**

The comparison was made with Wilcoxon signed-rank test and Chi-Square test. Correlations were analysed with the Pearson test. Statistical test was performed with the Statistical Packages for the Social Sciences (SPSS) for Windows version 25 (SPSS Inc., Chigaco, IL, USA). For the comparison between trauma grades, the Eshragi grades 1A and 1B were interpreted as grade 1 in micro-CT and histologic samples to have equivalent data with fusion images.

**Results**

In all TBs the insertion could be easily performed through the round window without any noticeable resistance during the insertion process. All insertions could be carried out easily, and there was no need to pull back and reinsert in any TB. Full insertion with all contacts inside the cochlea was achieved in 12 of the 14 TBs. Two electrode arrays exhibited partial insertions: In TB07 there was one extra-cochlear contact and in TB12 there were two extra-cochlear contacts. We did not notice any significant electrode bulging outside the cochlea in any of the insertions. The mean IDA measured from the CBCT images was 423 degrees (range 381 – 499 degrees) in all of the 14 TBs.

Trauma assessment via the CBCT fusion image technique showed scala dislocation in four TBs (TB02, TB05, TB07 and TB11; 29 %). In TB02 the electrode translocated occurred at IDA 360 degrees and the total insertion depth was 446 degrees. In TB05, TB07 and TB11 translocation occurred at IDA 180 degrees. The trauma grading is summarized in Table 1. Table 2 shows the comparison between trauma grading with the fusion imaging, micro-CT and histology.

TB07 and TB13 were excluded from the micro-CT and histological analysis due to displacement of the electrode arrays during the preparation of the specimen. For the remaining 12 TBs, both micro-CT and histology were of adequate quality and the Eshragi trauma scaling could be applied (image 1). In the micro-CT evaluation, trauma scaling for TB02 was class 3 (“translocation”), whereas in the histologic analysis trauma grading was class 2 (“rupture of basal membrane”). In the remaining TBs, trauma grading was identical for micro-CT and histology (Table 1).

There was no correlation between the IDA or trauma (r=0.216, p=0.458), A-measure and trauma (r=-0.462, p=0.093), or the basal length and trauma grading (p=0.220, r=0.450).

All of the methods provided similar results for trauma evaluation. There was a good agreement of trauma grading with all methods. The interpretation between micro-CT and histology was almost identical; a statistically significant difference could not be found (p=1.000). We found no statistical significance in trauma grading between either of the methods; fusion imaging and micro-TT (p=0.133) or the fusion imaging and histology (p=0.148).

**Discussion**

The electrode placement plays a crucial part in the success of CI surgery. During the development of new electrodes, histology has been the method for electrode placement evaluation in TB studies. Constant progress in imaging technology, accuracy and use of modern 3D reconstruction techniques are able to provide fast and reliable information regarding the intra cochlear placement of electrodes. However, applying the Eshragi trauma scaling in radiologic data is often challenging and thus, histology is still considered as the gold standard for trauma evaluation. In this study we aimed to develop more accurate imaging methods for evaluation of the insertion results in TB studies and exploit the possibilities of the 3D reconstructions.

Micro CT offers significant advantages over histology in TB studies: micro-CT is a non-destructive imaging method and considerably faster to perform (micro-CT 4-9 hours and histology approximately 90 days). In modern micro-CT devices detectors are even faster and more sensitive and hence imaging times can be even shorter. The development of more elaborated reconstruction algorithms also shortens the processing time of obtained images. Thus, accurate micro-CT images can be available in a few hours. Another advantage of the micro-CT is that the evaluation can be performed in a step-by-step ‘film strip’, following the whole length of electrode inside the cochlea (17). In addition, the micro-CT provides the possibility for 3D-reconstructions, from which the exact placement of electrodes inside the scala tympani can be seen more easily (Fig 2). Subtle anatomical variations in the cochlear duct, such as the so-called rollercoaster duct form, is only visible and recognizable from the 3D reconstructions. 2D images, histological or radiological, cannot convey that information.

However, we noted discrepancy between micro-CT and histology grading in TB02, in which micro-CT classified trauma as grade 3 (translocation), and histology as grade 2 (rupture of the BM). Yet, clinically both conditions could be regarded as inner ear trauma with probably similar clinical outcome as it is likely that in both situations any residual hearing would have been lost. The separation between the elevation of BM and rupture of BM with current imaging can be most challenging and even impossible, despite advanced reconstruction models (16) and image fusion.

Metal artefacts may reduce the micro-CT image quality and the artefact pattern is difficult to predict. Removing the perilymph prior to imaging improves the contrast between basilar membrane (BM), scala vestibuli and tympani, and aids the assessment of trauma with the CBCT image fusion technique and with micro-CT (20, 21). The micro-CT provides better resolution compared to CB-CT, but imaging time is longer than with CB-CT. The sample size with micro-CT is limited to smaller objects than with CB-CT, as well.

Earlier studies have shown that micro-CT is feasible for in-vitro electrode studies (15-17). Whereas Le Breton et al. (15) concluded that micro-CT is not sufficiently accurate for the reliable assessment of insertion trauma, Postnov et al. (17) found nearly equal visualization of the cochlear structures and the electrode array as in histologic samples. Similar to our study, Postnov et al. removed the perilymph to increase the contrast of the basilar membrane and better visualization. Teymori et al (16) found no difference in the electrode localization in 17 TBs assessed with micro-CT and histology. Teymori et al (16) classified electrode location as scala tympani if the array were fully inside the scala tympani, scala vestibuli if the electrode were above Reissner´s membrane and intermediate for electrodes at the scala media region. They also chose to use a grading differing from the Eshragi scale as in their 3D reconstruction model it was difficult to separate the elevation and rupture^16^.

The currently used trauma grading is based on histology and thus not directly applicable to radiologic evaluation. Therefore, a new trauma grading applicable for imaging should be developed. Mosnier et al. (9) and Seta et al. (8) used a simplified trauma staging (No trauma; dislocation) in their image-based studies. Accordingly, our study supports the feasibility of micro-CT for trauma assessment, with nearly the same accuracy as histology. However, there is a need for more validation studies to establish the value of micro-CT for electrode studies, as it has been rarely exploited in TB studies. To further evaluate the feasibility of micro-CT for CI TB studies, more research is needed including the development of a reliable trauma grading scale.

For the CBCT fusion image technique, differences in trauma grading are explained by the variety of the localization of the basilar membrane, which cannot be visualized with CBCT. Most of the differences were between grades 0 or 1. The clinical relevance of “no trauma” (grade 0) compared to the “touching of the basilar membrane” (grade 1) is not well distinct. There was only one single location in one TB when the CBCT image fusion technique graded the trauma as grade 1 compared to major trauma (grade 2 or 3) graded in the micro-CT and histology (TB02, 360 -degree point). Nevertheless, the overall trauma grade in TB02 was 3 for the image fusion technique as for micro-CT and histology it was 3 and 2 respectively. The accuracy of the image fusion technique has been documented in our previous studies (12, 13). This present study again verifies the overall good accuracy of the fusion image technique for TB studies, although the main advantage is its clinical feasibility.

With respect to the characteristics of the research electrode array, the trauma rate (REA) (29 % in fusion image and 25 % for micro-CT and histology, including the ruptured BM) is comparable to results obtained with the EVO electrode (13). The IDA for the REA in this study was almost identical with the previous study, 423 degrees (range 381-499) vs 416 degrees (range 368 to 501), respectively. Therefore, it appears that the increased basal stiffness did not facilitate deeper insertions. Even small alterations to the electrode array design, such as a slightly stiffer basal section, may influence in insertion properties of the electrode array. Thus, the suitability of new designs should be evaluated in TB studies in advance of clinical use.

Main limitations of this study are the small sample sizes. The use of single electrode is also a limitation, thus the different electrodes may cause different size of artefacts, so our findings have to be confirmed for other types of electrodes and manufacturers.

The strengths of this study are the availability of all three different methods for electrode location evaluation. Our institution has standardized methods for electrode studies and experience with different electrode characteristics. Thus, the presented results are comparable to previous studies.

**Conclusion**

This study supports the feasibility of micro-CT for trauma assessment, with nearly identical accuracy as histology. Although micro-CT provides very accurate information on the scalar location of the electrode, it is not sufficiently sensitive for the differentiation between basilar membrane elevation and rupture and therefore cannot completely substitute histology for the assessment of insertion trauma. The image fusion technique based on CBCT is less accurate as it relies on an empirical assumption of the basal membrane localization but it is clinically applicable.

**Legends**

Table 1. Descriptive data and overall trauma grading between different methods

Table 2. Distribution of insertion trauma with respect to different examination points regarding the insertion depth angle. FI = fusion image technique, µCT = micro-CT. TB02 and TB13 are excluded due missing µCT and histological results.

Figure 1. CB-CT 3D fusion, micro-CT and histologic images (1, 2 and 3 respectively) of TB 14 (A), TB 9 (B) and TB 2 (C). TB 14 shows no trauma, and TB 9 lifting at tip region (black arrow). TB 2 has dislocation in C1 and C2 images pointed by black arrow. In C3 the trauma was interpreted as rupture (pointed by black arrow).

Figure 2. Images from 3D-reconstructions videos of TB 14 (A1, A2 and A3), TB 9 (B1, B2 and B3) and TB 2 (C12, C2 and C3). Videos can be found from supplementary e-material, <http://links.lww.com/MAO/B576>, <http://links.lww.com/MAO/B577>, http://links.lww.com/MAO/B578.

Legends for the supplementary videos:

Supplementary video TB2: 3D reconstructed video from the TB2 based on the micro-CT scan. TB2 has a dislocation at 360-degree insertion depth angle.

Supplementary video TB9: 3D reconstructed video from the TB9 based on the micro-CT scan.

Supplementary video TB14: 3D reconstructed video from the TB14 based on the micro-CT scan.

REFERENCES

1. Aschendorff A, Kromeier J, Klenzner T, Laszig R. Quality control after insertion of the nucleus contour and contour advance electrode in adults. *Ear Hear*. 2007;28(2 Suppl):75S-79S. Accessed Mar 6, 2017. 10.1097/AUD.0b013e318031542e.

2. Holden LK, Finley CC, Firszt JB, et al. Factors affecting open-set word recognition in adults with cochlear implants. *Ear Hear*. 2013;34(3):342-360. https://www.ncbi.nlm.nih.gov/pubmed. Accessed Mar 6, 2017. 10.1097/AUD.0b013e3182741aa7.

3. Dhanasingh A, Jolly C. An overview of cochlear implant electrode array designs. *Hear Res*. 2017;356:93-103. Accessed Jun 7, 2019. 10.1016/j.heares.2017.10.005.

4. Wanna GB, Noble JH, Carlson ML, et al. Impact of electrode design and surgical approach on scalar location and cochlear implant outcomes. *Laryngoscope*. 2014;124 Suppl 6:1. Accessed Jun 7, 2019. 10.1002/lary.24728.

5. Buchman CA, Dillon MT, King ER, Adunka MC, Adunka OF, Pillsbury HC. Influence of cochlear implant insertion depth on performance: a prospective randomized trial. *Otol Neurotol*. 2014;35(10):1773-1779. https://www.ncbi.nlm.nih.gov/pubmed. Accessed Mar 6, 2017. 10.1097/MAO.0000000000000541.

6. Gantz BJ, Turner C, Gfeller KE, Lowder MW. Preservation of hearing in cochlear implant surgery: advantages of combined electrical and acoustical speech processing. *Laryngoscope*. 2005;115(5):796-802. Accessed Apr 30, 2018. 10.1097/01.MLG.0000157695.07536.D2.

7. Iso-Mustajärvi M, Sipari S, Löppönen H, Dietz A. Preservation of residual hearing after cochlear implant surgery with slim modiolar electrode. *Eur Arch Otorhinolaryngol*. 2020;277(2):367-375. Accessed Jul 13, 2022. 10.1007/s00405-019-05708-x.

8. De Seta D, Mancini P, Russo FY, et al. 3D curved multiplanar cone beam CT reconstruction for intracochlear position assessment of straight electrodes array. A temporal bone and clinical study. *Acta Otorhinolaryngol Ital*. 2016;36(6):499-505. Accessed Jul 8, 2020. 10.14639/0392-100X-1279.

9. Mosnier I, Célérier C, Bensimon J, et al. Cone beam computed tomography and histological evaluations of a straight electrode array positioning in temporal bones. *Acta Otolaryngol*. 2017;137(3):229-234. Accessed Jul 8, 2020. 10.1080/00016489.2016.1227477.

10. De Seta D, Torres R, Russo FY, et al. Damage to inner ear structure during cochlear implantation: Correlation between insertion force and radio-histological findings in temporal bone specimens. *Hear Res*. 2017;344:90-97. Accessed Jul 2, 2019. 10.1016/j.heares.2016.11.002.

11. Dietz A, Iso-Mustajärvi M, Sipari S, Tervaniemi J, Gazibegovic D. Evaluation of a new slim lateral wall electrode for cochlear implantation: an imaging study in human temporal bones. *Eur Arch Otorhinolaryngol*. 2018;275(7):1723-1729. Accessed Apr 21, 2020. 10.1007/s00405-018-5004-6.

12. Iso-Mustajärvi M, Matikka H, Risi F, et al. A New Slim Modiolar Electrode Array for Cochlear Implantation: A Radiological and Histological Study. *Otol Neurotol*. 2017;38(9):e327-e334. Accessed Oct 27, 2017. 10.1097/MAO.0000000000001542.

13. Sipari S, Iso-Mustajärvi M, Matikka H, et al. Cochlear Implantation With a Novel Long Straight Electrode: the Insertion Results Evaluated by Imaging and Histology in Human Temporal Bones. *Otol Neurotol*. 2018;39(9):e784-e793. Accessed Jun 22, 2019. 10.1097/MAO.0000000000001953.

14. Sipari S, Iso-Mustajärvi M, Löppönen H, Dietz A. The Insertion Results of a Mid-scala Electrode Assessed by MRI and CBCT Image Fusion. *Otol Neurotol*. 2018;39(10):e1019-e1025. Accessed Jun 22, 2019. 10.1097/MAO.0000000000002045.

15. Le Breton A, Jegoux F, Pilet P, Godey B. Micro-CT scan, electron microscopy and optical microscopy study of insertional traumas of cochlear implants. *Surg Radiol Anat*. 2015;37(7):815-823. Accessed Jul 13, 2022. 10.1007/s00276-015-1469-9.

16. Teymouri J, Hullar TE, Holden TA, Chole RA. Verification of computed tomographic estimates of cochlear implant array position: a micro-CT and histologic analysis. *Otol Neurotol*. 2011;32(6):980-986. Accessed Jul 13, 2022. 10.1097/MAO.0b013e3182255915.

17. Postnov A, Zarowski A, De Clerck N, et al. High resolution micro-CT scanning as an innovative tool for evaluation of the surgical positioning of cochlear implant electrodes. *Acta Otolaryngol*. 2006;126(5):467-474. Accessed Jul 13, 2022. 10.1080/00016480500437377.

18. Escudé B, James C, Deguine O, Cochard N, Eter E, Fraysse B. The size of the cochlea and predictions of insertion depth angles for cochlear implant electrodes. *Audiol Neurootol*. 2006;11 Suppl 1:27-33. https://www.ncbi.nlm.nih.gov/pubmed. Accessed Mar 6, 2017. 10.1159/000095611.

19. Eshraghi AA, Yang NW, Balkany TJ. Comparative study of cochlear damage with three perimodiolar electrode designs. *Laryngoscope*. 2003;113(3):415-419. https://www.ncbi.nlm.nih.gov/pubmed. Accessed Mar 6, 2017. 10.1097/00005537-200303000-00005.

20. Iso-Mustajärvi M, Sipari S, Lehtimäki A, Tervaniemi J, Löppönen H, Dietz A. A New Application of CBCT Image Fusion in Temporal Bone Studies. *J Int Adv Otol*. 2019;15(3):431-435. Accessed Jul 13, 2022. 10.5152/iao.2019.7365.

21. Avci E, Nauwelaers T, Lenarz T, Hamacher V, Kral A. Variations in microanatomy of the human cochlea. *J Comp Neurol*. 2014;522(14):3245-3261. Accessed Apr 21, 2020. 10.1002/cne.23594.
